# Genetic Manipulations of PPARs: Effects on Obesity and Metabolic Disease

**DOI:** 10.1155/2007/12781

**Published:** 2007-01-10

**Authors:** Yaacov Barak, Suyeon Kim

**Affiliations:** The Jackson Laboratory, 600 Main Street, Bar Harbor, ME 04609, USA

## Abstract

The interest in genetic manipulations of PPARs is as old as their discovery as receptors of ligands with beneficial clinical activities. Considering the effects of PPAR ligands on critical aspects of systemic physiology, including obesity, lipid metabolism, insulin resistance, and diabetes, gene knockout (KO) in mice is the ideal platform for both hypothesis testing and discovery of new PPAR functions in vivo. With the fervent pursuit of the magic bullet to eradicate the obesity epidemic, special emphasis has been placed on the impacts of PPARs on obesity and its associated diseases. As detailed in this review, understanding how PPARs regulate gene expression and basic metabolic pathways is a necessary intermediate en route to deciphering their effects on obesity. Over a decade and dozens of genetic modifications of PPARs into this effort, valuable lessons have been learned, but we are left with more questions to be answered. These lessons and future prospects are the subject of this review.

## 1. PPAR*α*

The only PPAR faithful to its acronym, PPAR*α*, is the nuclear receptor of peroxisome proliferators—a diverse group of compounds, which in addition to toxic and carcinogenic chemicals include the lipid-lowering fibrate drugs [1]. PPAR*α* is expressed in active metabolic tissues, including liver, heart, brown fat, and skeletal muscle, where it regulates genes that catalyze fatty acid (FA) catabolism [1, 2]. By 1995, mice homozygous for a *Ppara*-null allele were generated and found to be viable, healthy, fertile, and devoid of gross phenotypic defects under standard husbandry [3]. However, these mice could mount neither the hepatic response to peroxisome proliferators nor the induction of lipid-metabolizing enzymes by fibrates [3, 4]. These results confirmed the null nature of this allele, and obviated the need for alternative null configurations; consequently, this strain has become the exclusive animal model for studies of PPAR*α* deficiency to date. These studies are summarized below.

### 1.1. Ppara KO and obesity

Early studies of *Ppara*-null mice reported hepatosteatosis and elevated levels of circulating triglycerides (TG) and cholesterol, as well as a significant increase in gonadal fat pad mass [5–8]. The integral role of PPAR*α* in body fat mass determination is further cemented by the demonstration that the KO mice fail to decrease adipose tissue weight in response to hyperleptinemia [9]. However, the contribution of PPAR*α* to total body weight is ambiguous, with conflicting reports of substantial age-related obesity [6, 7] versus no significant body weight effects in congenic 129/SvJae or C57BL/6N *Ppara* ^−/−^ mice [8]. The discrepant outcomes of these studies have been attributed to subtle experimental variations in the genetic background and chow composition, and suggest that the contribution of PPAR*α* to obesity is strongly influenced by genomic and environmental contexts.

### 1.2. Ppara KO and fasting

The relatively minor phenotype of *Ppara*-null mice under standard husbandry conditions is consistent with a contingency function that comes into play under metabolic duress. Accordingly, multiple studies addressed the ability of *Ppara*-null to cope with dietary challenges. The most informative manipulation has been fasting, during which PPAR*α* deficiency was shown to cause excessive surge in circulating FA levels, rapid hepatic and cardiac lipid accumulation, absent ketogenic response, profound hypoglycemia and hepatic glycogen depletion [10–13]. These anomalies are thought to arise from failure of the mutant livers to catabolize adipose tissue-derived FA, which on the one hand impairs gluconeogenesis at both enzyme activity and substrate levels, and on the other hand leads to morbid accumulation of nonmetabolized lipids [14, 15]. PPAR*α* is similarly critical for cardiac lipid oxidation, which is the main energy source for the heart during fasting and exercise; reviewed in [16]. Both constitutive and inducible expression of PPAR*α* target genes are blunted in *Ppara*-null hearts, which exhibit abnormal TG accumulation during fasting and progressive deterioration of myofibrillar and mitochondrial integrity upon aging [10, 17]. The crucial importance of PPAR*α*-mediated hepatic and cardiac lipid catabolism, regardless of fasting, is also evident in the severe hypoglycemia and staggering lipid accumulation in livers and hearts of *Ppara*-null mice following pharmacological inhibition of FA flux [18]. Interestingly, female and estrogen-treated male *Ppara*-null mice are significantly protected against this lethal combination of tissue hyperlipidemia and systemic hypoglycemia, implying an alternative, estrogen-dependent lipid utilization pathway [18]. While proper cardiac metabolism is disrupted in the absence of PPAR*α*, dosage and temporal regulation of the receptor are critical, and its constitutive transgenic overexpression in cardiac muscle via the *α* myosin heavy chain (MHC) promoter is detrimental [19]. Hearts of *MHC-Ppara* transgenic mice exhibit a faithful phenocopy of diabetic cardiomyopathy, with increased lipid oxidation, a reciprocal decrease in glucose utilization, and symptoms of ventricular hypertrophy [19].

### 1.3. Ppara KO in high-fat and cholesterol-rich diets

The role of PPAR*α* in the physiological outcomes of high fat diet (HFD) is not as clear as its role in fasting physiology. *Ppara*-null mice are as susceptible as *wt* mice to HFD-induced weight gain and hepatic TG accumulation, but are protected from glucocorticoid-induced hypertension [20–22]. Blunted hyperinsulinemia and improved glucose and insulin tolerance following 2-hour fasting suggested initially that HFD-fed *Ppara*-null mice are protected from insulin resistance (IR) as a result of either reduced hepatic glucose production or increased peripheral insulin sensitivity [20]. However, this report has been contested by a study that found little difference in hyperinsulinemia and peripheral glucose uptake during euglycemic clamp of HFD-fed *wt* versus *Ppara*-null mice in the nonfasted state [23]. This contradictory result raised the concern that the improved insulin and glucose tolerance of HFD-fed *Ppara*-null mice in the earlier studies reflects no more than their established hypoglycemic response to the fasting regimen that preceded the assays; studies that bypass this conceptual hurdle will be required to reevaluate the role of PPAR*α* in the aftermath of HFD. Because the consequences of PPAR*α* deficiency also include a constitutive increase in circulating cholesterol, it is equally important to test the effects of a cholesterol-rich diet in *Ppara*-null mice. Surprisingly, addition of 2% cholesterol to the chow caused fat pad weight reduction and increased de novo lipogenesis in *Ppara*-null mice, indicating that the receptor participates in basal and feedback-regulated cholesterol and triacylglycerol homeostasis in adipose tissue [24]. These activities impinge directly on the contribution of PPAR*α* to obesity.

### 1.4. Effects of Ppara KO on other tissues

Unlike the effect of PPAR*α* on cardiac muscle, PPAR*α* deficiency had no significant effect on the responses of skeletal muscle to either fasting or heavy exercise, perhaps due to compensation by redundant functions of PPAR*δ* [25]. However, transgenic overexpression of PPAR*α* in skeletal muscle, using the muscle creatine kinase (MCK) promoter, protected mice from HFD-induced obesity, albeit at the expense of glucose intolerance and insulin resistance [26]. The proposed mechanism entails reduced insulin-stimulated glucose uptake due to repression of AMP-activated protein kinase-dependent glucose transporter gene expression by oxidized FA. The hepatocentric view of systemic PPAR*α* effects is moderated by a recent report of increased peripheral glucose utilization in fasted *Ppara*-null mice, which was refractory to adenovirus-mediated reconstitution of hepatic PPAR*α* [27]. Moreover, direct injection of a PPAR*α* agonist into the lateral ventricle of *wt* mice significantly reduced whole body glucose utilization, suggesting that PPAR*α* also functions centrally [27]. Tissue-specific *Ppara*-null mice, which have yet to be generated, would be an ideal platform to validate and further explore these intricate mechanisms of PPAR*α* action.

## 2. A PANOPLY OF PPAR*γ* KNOCKOUTS

Without detracting from the importance of PPAR*α* and PPAR*δ* (see below for PPAR*δ*), the defining moment in the explosive growth of the PPAR field has been the identification of PPAR*γ* as the high-affinity receptor of the insulin-sensitizing thiazolidinedione (TZD) drugs [28, 29]. The pivotal role of PPAR*γ* in the adipocyte life cycle [30–34], combined with the blockbuster success of its TZD ligands in treating type II diabetes [35, 36], generated widespread enthusiasm for the prospect of solving the causal relationship between obesity and diabetes through PPAR*γ* research. The use of gene knockout in mice presented the most logical investigative approach, leading to the generation of a dazzling array of mouse strains with genetic modifications of *Pparg*. This volume of effort reflects not only the immense biomedical significance of the gene, but also the complexity of the genetic data, which had encumbered immediate, straightforward understanding of PPAR*γ* function in vivo and had spawned numerous alternative hypotheses. The myriad *Pparg* KO strains, and the results of their analyses, are summarized below.

Constitutive Pparg deficiency cannot be studied in adult mice due to the essential role of PPAR*γ* in placental development, which abolishes survival beyond mid-gestation [34]. However, aggregation with tetraploid *wt* embryos provided *Pparg*-null embryos with *wt* placentas and rescued them to term [34]. In these chimeras, *Pparg*-null cells committed to the adipocyte lineage, but failed to proliferate and differentiate into *bona fide* adipocytes, and a chimeric pup that survived a few days after birth was devoid of any type of adipose tissue [34]. This effort proved the essential role of PPAR*γ* in early adipogenesis in vivo, but unfortunately, perinatal lethality precluded studies of this *Pparg*-null configuration beyond birth. The current availability of floxed *Pparg* alleles (see below) and epiblast-specific Cre-expressing mouse strains [37, 38] should revitalize this configuration by facilitating higher throughput generation of *Pparg*-null mice supported by *wt* placentas. Studies in progress in our lab with standard *wt/Pparg*-null chimeras, in which diploid host-derived *wt* cells coexist with *Pparg*-null ES-derived cells, confirmed the formation and subsequent arrest of *Pparg*-null adipose tissue primordia. However, here *wt* cells infiltrated the stagnant *Pparg*-null primordia and repopulated them through a previously unknown developmental feedback mechanism (S. Kim and Y. Barak, unpublished). Consequently, post-term *wt/Pparg*-null chimeras invariably possess only *wt* adipose tissue, in contrast to the random contribution of *wt* and *Pparg*-null cells to other tissues [31].

### 2.1. Pparg^+/−^mice

With *bona fide* adult *Pparg*-null mice unavailable, investigators initially turned to *Pparg*-haploinsufficient mice to explore the effects of reduced PPAR*γ* dosage. As expected, adiposity of *Pparg* ^+/−^ mice was reduced, supporting the assertion that PPAR*γ* contributes quantitatively to adipose mass [39]. However, contrary to the expectation that reduced levels of the TZD receptor will cause a parallel reduction in insulin sensitivity, *Pparg* ^+/−^ mice were more insulin-sensitive than *wt* controls when challenged by either HFD or aging [39–41]. This confounding observation conflicts with the monochromatic view of PPAR*γ* as a beneficial TZD-activated insulin sensitizer and raises the counterintuitive notion that it has pathogenic activities. While the nature of these adverse properties of the receptor is unclear, one potential example of a latent pathogenic effect is the positive relationship between PPAR*γ* dosage and adipose tissue mass, which might come into play under conditions of nutritional affluence. However, excessive reduction of PPAR*γ* activity by treating haploinsufficient mice with a PPAR*γ* antagonist reversed the tide and resulted in lipodystrophy and IR [42, 43].

### 2.2. Tissue-specific Pparg KOs

Tissue-specific Pparg KOs were subsequently developed by several groups using *Cre-loxP* methodology, with the vision of both bypassing the embryonic lethality of constitutive *Pparg* deficiency and resolving the physiological functions of PPAR*γ* one tissue at a time [32, 44, 45]. *Pparg* has since been deleted in a substantial number of cell types, of which the most pertinent to this review are adipocytes, myocytes, and hepatocytes, and from a broader metabolic disease perspective also macrophages, pancreatic *β*-cells, renal collecting duct epithelia, and endothelial cells (referenced below).

#### 2.2.1. Adipocyte-specific Pparg KO

The abundant expression of PPAR*γ* in adipocytes indicates that its important functions in these cells extend beyond its indispensability for their formation. Moreover, the association between obesity as well as type II diabetes and the antidiabetic effect of TZDs fuel the hypothesis that PPAR*γ* activity in adipocytes is a key to systemic insulin sensitivity. A mouse whose adipocytes lack PPAR*γ* would provide the ultimate test for this idea. Generation of such a model was attempted using an adipocyte-specific *Fabp4*(aP2)-CRE transgene. While, as mentioned earlier, PPAR*γ* is essential for adipocyte differentiation, the *Fabp4* promoter is activated after completion of adipogenesis, and thus allows the PPAR*γ*-dependent formation of adipocytes prior to *Pparg* deletion [32, 45]. Contrary to a widespread, unsubstantiated concern, the *Fabp4* promoter does not drive transgene expression in macrophages or other major metabolic tissues [32], and therefore the phenotype of these mice is not muddled by gene deletion in nonadipocyte cell types. Adipocyte-specific *Pparg*-null mice exhibited rapid loss of brown adipose tissue (BAT) and subcutaneous fat [32, 45]. Astonishingly, however, white adipose tissue (WAT) retained normal mass throughout a substantial stretch of adulthood [32]. This retention occurred despite substantial cell death and extensive fibrosis and inflammatory infiltration, and resulted from both overt hypertrophy of surviving adipocytes and adipocyte regeneration [32, 45].

The tight dependence of adipocytes on PPAR*γ* for survival and the interpretation that adipocyte regeneration mitigates lipodystrophy were unequivocally proven by studies of mice with tamoxifen-inducible adipocyte *Pparg* KO [33]. These mice carry a *loxP*-flanked (floxed) *Pparg* allele and an *Fabp4*-driven fusion of Cre with a tamoxifen-responsive estrogen receptor mutant, which translocates to the adipocyte nucleus and targets the floxed allele only in response to tamoxifen administration. Induction of Cre activity in these mice induced synchronous, near-complete loss of white and brown adipocytes within 7 days, followed by acute inflammatory infiltration of the damaged fat pads, and complete rebound of adipocyte number and adipose tissue integrity within 6 weeks of the initial insult [33]. Thus, PPAR*γ* is essential for the survival of mature adipocytes, but a rapid and robust regenerative process mitigates a loss of fat tissue following *Pparg* deletion. Similar regenerative potential of adipose tissue was recently demonstrated in mice with inducible adipocyte apoptosis [46], buttressing the notion that fat regenerates with remarkable efficiency in response to adipocyte death, beyond the context of PPAR*γ* deficiency. Thus, adipose tissue of *Fabp4*-Cre *Pparg* ^fl/fl^ mice comprised a dynamic mixture of dying *Pparg*-null adipocytes alongside repopulating *wt* adipocytes—a condition that hindered the generation of mice with adipose tissue that uniformly lacks PPAR*γ*. As long as WAT was sustained, these mice maintained relatively normal lipid and glucose homeostasis, despite substantial reduction in circulating leptin and adiponectin and an anticipated rise in the levels of free FA [32, 45]. Moderate IR and glucose intolerance, as well as hepatomegaly, steatosis, and increased hepatic glucose production, developed only in conjunction with the eventual terminal atrophy of WAT [32]. Analyses of two independent stocks of these mice by two research teams found obvious resistance to HFD-induced obesity, likely due to the failure to accumulate adipocytes. However, the two teams observed opposite effects on insulin sensitivity. In one study, HFD accelerated lipoatrophy and exacerbated IR [32], whereas the other study found no such degenerative effect and the mutation protected the mice from IR [45]. In summary, while reproving the critical role of WAT in systemic insulin sensitivity and the indispensability of PPAR*γ* to adipocyte viability, the adipocyte-specific *Pparg*-null mouse fell short of a definitive demonstration that adipocyte PPAR*γ* regulates whole body metabolism.

#### 2.2.2. Myocyte-specific Pparg KO

The insulin sensitizing activity of PPAR*γ* ligands and the key role of skeletal muscle in peripheral insulin sensitivity generated great interest in the hypothesis that PPAR*γ* exerts its insulin sensitizing activity from within myocytes. However, this hypothesis was challenged by the very low basal expression of PPAR*γ* in skeletal muscle. The issue was addressed by two parallel studies that analyzed the outcome of *Pparg* deletion in myocytes. In the first study, myocyte-specific *Pparg*-null mice generated by *MCK* promoter-driven Cre recombinase exhibited increased adiposity, elevated susceptibility to HFD-induced weight gain, and marked hepatic IR in hyperinsulinemic-euglycemic clamps [47]. However, these mice were only as sensitive to HFD-induced IR and as responsive to the insulin sensitizing effects of TZDs as *wt* mice, suggesting that muscle PPAR*γ* is dispensable for the antidiabetic effects of PPAR*γ* agonists. The second study used mice generated using the same Cre transgene, but a different floxed *Pparg* allele, and first addressed the controversial issue of low PPAR*γ* expression in myocytes [48]. It demonstrated that the minute amount of *Pparg* mRNA observed in muscle extracts undergoes *MCK*-Cre-mediated recombination, and thus, unequivocally proved that the transcript originated in myocytes rather than other cell types that populate muscle tissue. Mice in this study developed insulin and glucose intolerance with age, and exhibited severely compromised insulin-stimulated muscle glucose uptake, as well as liver and adipose tissue IR. In contrast to the first study, here TZDs failed to ameliorate muscle insulin resistance, suggesting that myocyte PPAR*γ* regulates muscle insulin sensitivity cell autonomously. While the differential sensitivity of the two strains to TZDs raises concerns about the validity of the interpretations, they are not necessarily contradictory, considering that both the metabolic challenges (HFD versus aging) and the assayed activities (Insulin tolerance tests versus muscle glucose uptake) were different in each study. Still, more definitive studies, using mice with a purer genetic background and a standardized experimental approach, are required to settle these discrepancies. Regardless of the final answer, it is clear that while PPAR*γ* may have some metabolic functions in myocytes, these functions are not sufficiently robust to account for the systemic antidiabetic actions of TZDs.

#### 2.2.3. Hepatocyte-specific Pparg KO

As in muscle, basal PPAR*γ* expression in liver is minimal. However, hepatic PPAR*γ* expression is induced substantially during steatosis. The effects of albumin Cre-mediated hepatocyte *Pparg* deficiency were studied in *wt* and two different diabetic mouse models that succumb to steatosis—*A-Zip/F* lipoatrophic mice and leptin-deficient *ob/ob* mice [49, 50]. On an otherwise *wt* background, hepatic *Pparg* deficiency caused a significant defect in TG clearance, hyperlipidemia, and increased body fat mass with age, demonstrating the importance of hepatic PPAR*γ* for basal fat tolerance and management of adiposity [50]. On the two diabetic backgrounds deficiency of hepatocytes for *Pparg* caused marked amelioration of hepatosteatosis, but exacerbated hyperlipidemia and muscle insulin resistance [49, 50]. These traits were reversed by TZDs in *ob/ob*, but not A-Zip/F mice, suggesting that the drugs exert their effect through activation of PPAR*γ* in adipocytes, not hepatocytes. Together, these studies indicate that hepatocyte PPAR*γ* is required for basal fat tolerance and, in addition, for steatosis of the diabetic liver, which serves to improve TG homeostasis and dampen systemic IR. However, they also clearly indicate that hepatic PPAR*γ* is not critical for TZD-induced insulin sensitization.

#### 2.2.4. Other tissue-specific Pparg KOs

The relatively modest effects of PPAR*γ* deficiencies in fat, muscle, and liver provided the impetus for broadening the analysis of *Pparg* KO to additional cell types that participate in obesity-associated metabolic complications, namely diabetes, hypertension, and atherosclerosis. The outcomes of these analyses are briefly summarized as follows.

*Pparg* deficiency in *β*-islets caused a hyperplastic response without altering glucose homeostasis, ruling out a critical function of the receptor in homeostatic functions of *β* cells [51].

A strong rationale for the generation and analysis of *Pparg*-null macrophages was provided by observations that TZDs induce macrophage genes that regulate lipid flux, suppress inflammatory gene expression, and ameliorate atherosclerosis [52–54]. Early studies with *Pparg*-null macrophages in culture and in vivo demonstrated that TZD effects on lipoprotein flux indeed depend on PPAR*γ*, but several of the reported anti-inflammatory effects of TZDs are independent of PPAR*γ* [44, 55]. Nevertheless, adaptive transfer of *Pparg*-deficient macrophages exacerbated genetic- and diet-induced atherosclerosis in recipient mice, demonstrating that PPAR*γ* performs key anti-atherogenic functions in these cells [56, 57]. In addition, a recent, yet-to-be-published symposium talk reported that macrophage-specific *Pparg*-null mice are glucose intolerant and exhibit increased sensitivity to HFD-induced insulin resistance [58]. Thus, PPAR*γ* orchestrates multiple beneficial activities in macrophages that could be harnessed for the development of advanced therapies for atherosclerosis.

Edema due to fluid retention is an undesired side effect of TZD treatment in diabetic patients [59]. Mice with *Pparg* KO in renal collecting duct epithelia are resistant to this TZD-borne complication, confirming that PPAR*γ* mediates it, apparently by enhancing sodium retention [60, 61]. This activity highlights an additional mechanism through which PPAR*γ* may regulate plasma volume, hypertension, and cardiovascular function.

*Pparg* deletion in endothelial cells exacerbates both HFD-induced and salt-induced hypertension, and renders the condition nonresponsive to TZDs, demonstrating that endothelial PPAR*γ* is critical for mitigating the effects of dietary stress on blood pressure [62].

Ablation of PPAR*γ* in cardiomyocytes causes elevated cardiac NF-*κ*B activity and increased expression of cardiac embryonic genes, which lead to enhanced myofibril assembly and cardiac hypertrophy but does not affect systolic function [63]. The relationship between this phenotype and the metabolic functions of PPAR*γ* in other tissues is not entirely clear, although aspects of cardiac lipid metabolism have yet to be addressed in this mouse.

### 2.3. Pparg2-specific KOs

Alternative promoters give rise to several *Pparg* isoforms with distinct 5′ ends. PPAR*γ*1 is the ubiquitous isoform, expressed in all PPAR*γ*-expressing tissues [64]. Adipocytes express, in addition to PPAR*γ*1, a cell-specific isoform termed PPAR*γ*2, whose unique 5′ exon encodes a 30 residue-long N-terminal extension of the ligand-independent transactivation domain of PPAR*γ*1 [65]. Because the placenta expresses only PPAR*γ*1, KO of PPAR*γ*2 could provide yet another means to bypass the lethal outcome of constitutive *Pparg* deficiency, as well as to interrogate potential unique functions of this adipocyte-specific isoform. In all, three teams have knocked out *Pparg2* using distinct targeting strategies that produced slightly different results [66–68]. Knock-in of red fluorescent protein into the *Pparg2*-specific B exon produced a clean KO of PPAR*γ*2 while retaining normal PPAR*γ*1 expression in adipocytes [66]. This configuration interfered with adipocyte differentiation in vitro and markedly reduced fat mass in vivo. This lipodystrophic phenotype involved significant reduction in the size, number, and TG content of brown and white adipocytes, and decreased expression of typical adipocyte markers [66]. A second knockout configuration entailed replacement of the entire B exon and flanking intronic sequences with a *lacZ-neo* cassette and resulted in a similar *Pparg2*-specific gene disruption, without affecting *Pparg1* [67]. This configuration was as detrimental to adipocyte differentiation in vitro as the previous KO configuration, but unlike that KO it had only a marginal effect on either fat mass or basal adipocyte size [67]. It is unclear whether these differences are meaningful or rather reflect minor differences in the experimental setup used by the two teams, for example in allele configuration, the genetic background of the mice, composition of the chow, or analytical methods. A third *Pparg2* targeting configuration, which resulted from an intronic neo cassette downstream of exon B, eliminated *Pparg2* expression but inadvertently altered *Pparg1* expression, abolishing it in WAT while augmenting it in BAT [68]. Mice homozygous for this modification were deemed PPAR*γ* hypomorphs (*Pparg* ^hyp/hyp^). Unlike the first two configurations, *Pparg* ^hyp/hyp^ were subject to high mortality rate and growth retardation during infancy; survivors thrived after weaning but were substantially lipodystrophic [68]. Importantly, contrary to other models of lipodystrophy, all three *Pparg2*-null configurations, including *Pparg* ^hyp/hyp^, exhibited a surprisingly modest decrease in glucose or insulin tolerance and did not develop steatosis. The suggestion that this relatively healthy phenotype is mitigated by compensatory lipid oxidation in muscle tissue [68] has to be reconciled with the failure of a similar compensatory mechanism to offset other cases of lipodystrophy.

### 2.4. Knock-in of dominant-negative mutations from human patients

As if the analyses described to this point were not sufficiently counterintuitive and indecisive, mice heterozygous for *Pparg-L466A* or *Pparg-P465L*—two dominant-negative missense mutations identified in human subjects—provided further surprises. Patients carrying one allele of either mutation alongside a second *wt* allele suffer from partial congenital lipodystrophy with hallmarks of the metabolic syndrome, including dyslipidemia, early-onset type II diabetes, and hypertension [69–73]. It therefore made perfect sense to replace the mouse *Pparg* gene with similar mutations, with the obvious expectation of recapitulating the clinical phenotype. Two research teams carried out this endeavor, each knocking in one of the mutations [74, 75]. Mice homozygous for either mutation died in utero, demonstrating the null nature of the alleles. However, while mice heterozygous for either mutation exhibited moderate hypertension and anomalies of either fat distribution or adipocyte morphology, none fully recapitulated the lipodystrophic phenotype of the orthologous patients [74, 75]. Moreover, *Pparg* ^*P465L/+*^ mice displayed no gross changes in plasma chemistry and were in fact more glucose tolerant than *wt* mice, both basally and following HFD, just like standard *Pparg*-haploinsufficient mice [74]. In addition, although more physiological anomalies were reported for *Pparg* ^*L466A/+*^ mice compared to *Pparg* ^*P465L/+*^ mice, including elevated free FA levels, hepatic steatosis and HFD-induced insulin resistance [75], their morbidity did not amount to that of their human counterparts.

### 2.5. Other genetic manipulations of Pparg

In addition to the *Pparg* KO onslaught, there has been a substantial public health interest in more subtle aspects of its function. These include the effects of genetic polymorphisms and post-translational modifications, which have been linked both genetically and epidemiologically to obesity and type II diabetes in the human population [76–79]. The first reported effort that undertook this approach is the S112A point mutation, which eliminates a MAP kinase phosphorylation site that inhibits the transcriptional activation capacity and adipogenic functions of PPAR*γ* [80]. *Pparg* ^*S112A/S112A*^ mice are viable and healthy, and do not display physiological anomalies under normal husbandry. However, the failure to regulate PPAR*γ* action by phosphorylation protects these mice against HFD-induced adipocyte hypertrophy and insulin resistance [80]. These results validate the utility of subtle structural mutations for uncovering important physiological activities of PPAR*γ*. Informal communications with other researchers, as well as the public NIH grant database, reveal that additional genetic manipulations aimed at understanding the biological function of conserved and polymorphic sequence elements of PPAR*γ* are currently underway in mice.

### 2.6. Pparg KOs—summary and remarks

In aggregate, a slew of attempts to generate molecular genetic models that will reveal a role for PPAR*γ* in obesity, insulin resistance, and related metabolic disorders have yielded partial success and confounding results. Constitutive *Pparg* KO was nonviable, *Pparg* haploinsufficiency was unexpectedly beneficial, and the pathogenic effect of dominant-negative *Pparg* mutations in human patients was not faithfully replicated in mouse models. Reassuringly, chimeric mouse studies and adipocyte-specific KOs unequivocally proved the critical role of PPAR*γ* in adipocyte differentiation and survival. However, the potential for an interpretable effect on energy metabolism was thwarted by the inability to obtain long-lasting *Pparg*-null adipocytes, which did not allow teasing out the effect of PPAR*γ* deficiency from the general impact of lipodystrophy. Quite disappointingly, KOs in other tissues had relatively modest effects basally and latent metabolic defects in response to dietary or genetic challenges. While these studies invoked encouraging links to atherosclerosis and hypertension, none amounted to full-blown IR, let alone diabetes. These major deviations from straightforward expectations raise concerns about the applicability of genetic studies of *Pparg* in the mouse to human metabolism. However, one should be reminded that TZDs are equally potent as insulin sensitizers in both mice and humans [35, 36], highly suggestive of similar metabolic functions of PPAR*γ per se* across species. A more likely explanation for the relatively benign outcomes of these studies is the inherently fickle nature of genetic, physiological, and metabolic experiments in mice. Evolution likely differentiated metabolic physiology in rodents versus humans, and although PPAR*γ* may have the exact same function in the bigger scheme, other genes and pathways may modify the outcome. In addition, lab mice are reared in a highly controlled ambient and provided either with uniform lean chow that differs drastically from human diet, or with experimental diets that mimic our own dietary follies, but which rodents have not evolved to handle. Effects of genetic background and modifier genes on outcomes and their interpretation comprise another obstacle. On the one hand, many of the studies summarized here do not clarify the extent of genetic homogeneity of the tested cohorts, potentially obstructing minor, yet critical effects of the mutations. On the other hand, the human population is genetically diverse, and gene defects that would devastate one person could be inconsequential in another. A case in point is the dramatic exacerbating effect of a mutation in the PPP1R3A gene on the outcome of PPARG mutations in a human pedigree [72]. Genes and pathways with comparable modifying effects could compensate for the effects of *Pparg* deficiency in mice. Moreover, redundant activities of PPAR*γ* in different tissues or an altogether misguided choice of target tissues and readouts might have further hindered interpretation. Finally, it may be time to start entertaining the notion that the problem might be with the hypothesis itself: clearly, activation of PPAR*γ* with TZDs is a robust therapy for IR, but does this mean that the pathway is necessary for basal insulin sensitivity in mice and men?

## 3. PPAR*δ*

PPAR*δ* was initially regarded as a promising prospect for studies of obesity and associated diseases purely on the merit of its pharmaceutically accomplished homologues [64]. With pharmacological agonists and genetic manipulations of PPAR*δ* coming to fruition in recent years, these expectations are starting to be realized, and implicate PPAR*δ* in important aspects of obesity, energy metabolism and metabolic disease. As in *Pparg*-null mice, analysis of *Ppard* deficiency also faces the challenge of substantial embryonic mortality, albeit for completely different reasons. The nature of the challenge, the different solutions, and the associated caveats are discussed briefly as a primer to the review of phenotypes associated with *Ppard*-null and gain-of-function models.

In all, 6 *Ppard*-null configurations have been generated in mice. Three knockout strains harboring deletions or insertions that wipe out the PPAR*δ* protein product in its entirety cause severe placental defects that lead to substantial embryonic mortality [81–83]. While there are practically no surviving homozygous null animals on the standard, C57BL/6 (B6) background, survival is increased to between 5% and 20% on outbred B6 : 129/Sv [81] or FVB : B6 backgrounds (Y. Barak, unpublished data). Unfortunately, *Ppard*-null mice and *wt* controls generated in this fashion inherently possess mixed genetic backgrounds, whose stochastic quantitative trait locus effects significantly muddle physiological data. In addition, through successive interbreeding of surviving homozygous null FVB : B6 mice over several generations, our lab has managed to generate a genetically semistable *Ppard*-null stock with approximately 50% survival (Y. Barak, unpublished data). However, while this stock provides a higher yield of *Ppard*-null mice with a relatively isogenic background, the nature of the breeding strategy hindered the generation of genetically matching *wt* controls. In a fourth *Ppard* null allele, no substantial embryonic lethality was reported [84]. However, in this allele *Ppard* was truncated 60 amino acids from its C-terminus, leaving its entire DNA-binding domain and most of its ligand-binding domain intact, and raising a reasonable concern that it is a hypomorph that enabled embryonic survival via residual PPAR*δ* functions. Therefore, analyses of adult mice carrying this KO configuration have to be interpreted with the cautionary note that it is likely incompletely deficient for PPAR*δ*. Finally, floxed *Ppard* alleles have been generated as well [81, 85]. These configurations enable the targeting of *Ppard* in specific tissues with the obvious caveat that Cre-mediated deletion of floxed alleles is seldom fully penetrant. To avoid confusion, the term *Ppard*-null mice is used in the following text to describe animals with germ-line deletion of the gene in all tissues, whereas studies performed with tissue-specific *Ppard* KOs are spelled out.

### 3.1. Genetic manipulations of PPAR*δ* and adipose tissue

Early studies of outbred *Ppard*-null mice under standard husbandry conditions revealed a substantial decrease in the size of BAT and WAT [81, 84]. Fat mass was not reduced in adipocyte-specific *Ppard*-null mice (floxed *Ppard x Fabp4-Cre*) [81], demonstrating that this trait is not adipocyte-autonomous, and must result from impaired PPAR*δ* activity in other tissues. While unable to achieve normal adiposity on standard, low fat chow, *Ppard*-null mice underwent a quicker and substantially more aggressive weight gain in response to HFD compared to *wt* controls [86, 87]. These observations were complemented and extended by studies of mice expressing constitutively active PPAR*δ* in adipose tissue [87]. In these mice, the *Fabp4* promoter drives adipocyte-specific expression of a fusion protein between the transactivation domain of the Herpes Virus VP16 protein and PPAR*δ* (*Fabp4-VP-Ppard*), such that the latter is rendered permanently active, irrespective of endogenous ligands. When reared on standard, low-fat chow *Fabp4-VP-Ppard* mice exhibited significant reduction in body weight and in the overall mass and TG content of adipose tissue, as well as in the levels of circulating TG and free FA [87]. However, the mice were protected from the adipocyte hypertrophy, dyslipidemia, obesity, and steatosis that occur in response to either HFD or impaired leptin signaling [87]. Quelling of obesity in these mice was associated with upregulation of genes that control lipid catabolism and adaptive thermogenesis in both BAT and WAT; reassuringly, the same genes are induced in response to systemic administration of a PPAR*δ* ligand [87]. In contrast, adipocyte-specific PPAR*δ* deficiency compromised HFD-mediated induction of the uncoupling protein 1 gene, *Ucp1*, in BAT [87]. Combined, these two genetic extremes of deficiency versus constitutive activation identify PPAR*δ* as a critical regulator of lipid homeostasis and adiposity.

### 3.2. Genetic manipulations of PPAR*δ* and muscle

The abundant expression of PPAR*δ* in myocytes suggests an important role in skeletal muscle [2]. Two transgenic models of muscle-specific PPAR*δ* overexpression and one of muscle-specific *Ppard*-deficiency confirmed this notion and revealed a massive impact of PPAR*δ* on muscle and whole body physiology. MCK promoter-driven expression of constitutively active *VP-Ppard* resulted in a dramatic type switch of muscle from type II, glycolytic fibers to type I, slow-twitch, oxidative fibers, and a staggering increase in aerobic endurance [88]. This switch was associated with activation of the typical oxidative fiber expression program, including genes that regulate lipid catabolism, mitochondrial electron transfer, oxidative metabolism, and type I contractile structures [88]. Overexpression of *wt Ppard* in skeletal muscle activates a similar expression pattern, and falls just short of inducing fiber-type switching [89]; the tamer induction of these genes in the latter mouse strain reflects the lesser activity of *wt* PPAR*δ* compared to the VP16-fused variant. These observations were fully corroborated by skeletal muscle-specific KO of *Ppard*, which resulted in the reciprocal muscle type switch from high- to low-oxidative fibers [85]. Molecular analyses of these mice revealed that PPAR*δ* regulates the expression of the transcriptional cofactor PGC1*α*, which regulates mitochondrial biogenesis and muscle type switch, providing a plausible mechanistic explanation for the basis of PPAR*δ* function in muscle [85]. Remarkably, constitutive activity of PPAR*δ* in muscle protected the mice from HFD-induced adipocyte hypertrophy, obesity, and IR, demonstrating the major influence of PPAR*δ*-induced energy dissipation in muscle on systemic energy homeostasis [88]. In full agreement with these findings, muscle-specific *Ppard* deficiency resulted in obesity, adipocyte hypertrophy, and insulin resistance [85]. Moreover, the basal respiratory quotient and glucose tolerance of whole-body *Ppard*-null mice are significantly reduced in the absence of additional dietary or genetic challenges [90]. Combined, these observations indicate that enhancement of basal metabolism by PPAR*δ* in general, and in muscle in particular, are critical for systemic energy homeostasis, and play a pivotal role in curbing obesity and its metabolic sequelae.

In addition to the gain and loss-of-function studies in skeletal muscle, loss-of-function studies revealed a critical requirement for PPAR*δ* also in cardiac muscle. Cardiomyocyte-specific *Ppard*-null mice (floxed *Ppard x MHC-Cre*) exhibited reduced expression of genes regulating FA oxidation, accompanied by progressive cardiac lipid accumulation, cardiac hypertrophy, and dilated cardiomyopathy [91]. The mice develop typical symptoms of congestive heart failure and died within the first 10 months of life, demonstrating the vital importance of PPAR*δ* for myocardial FA oxidation and function [91]. Considering that mice carrying germ-line *Ppard* deficiency reach old age without major incident [81, 85], the harsher phenotype of mice that lack this PPAR only in the heart requires explanation. In addition, as PPAR*α* induces similar pathways of cardiac FA oxidation and protection from lipotoxicity (see above), it will be crucial to determine how these differ from those regulated by PPAR*δ*, and why neither PPAR compensates for the deficiency of the other.

### 3.3. Genetic manipulations of PPAR*δ* and atherosclerosis

The abundant expression of PPAR*δ* in macrophages provided a compelling rationale to study its contribution to macrophage biology and atherosclerosis. Comparative studies of *wt* versus *Ppard*-null embryonic stem cell-derived macrophages identified very low-density lipoprotein (VLDL) as a rich source of PPAR*δ* agonists and the gene for the lipid droplet-associated ADRP protein as a tightly regulated PPAR*δ* target gene [82]. Combined with the observed increases in hepatic VLDL production, circulating VLDL levels, and VLDL-associated TG in *Ppard*-null mice [86], this functional interaction suggested that PPAR*δ* is engaged in negative feedback regulation of systemic VLDL flux. While these studies provide circumstantial support for the potential role of PPAR*δ* in macrophage lipid metabolism, subsequent studies found no effect of PPAR*δ* deletion or activation on cholesterol flux in macrophages [92]. In contrast, deletion of the *Ppard* gene reduced the expression of pro-inflammatory genes in macrophages, as did treatment with PPAR*δ* agonists [92]. The similar effects of PPAR*δ* deficiency and activation invoke a mechanism, in which the association of unliganded PPAR*δ* with transcriptional corepressors promotes inflammation, which can be relieved by either ligand-mediated derepression or an outright gene KO. Most importantly, these activities have a measurable impact on atherosclerosis, and transplantation of *Ppard*-null bone marrow markedly suppressed atherosclerosis in LDL-receptor KO mice [92]. Thus, basal PPAR*δ* activity in macrophages augments the pathogenesis of atherosclerosis, and PPAR*δ* ligands may exert therapeutic effects by reversing, rather than enhancing, this pathogenic activity.

## 4. SUMMARY AND PROSPECTS

This review summarized the insights obtained into the functions of PPARs in obesity and metabolic disease through genetic manipulation of mice. For focus purposes, we excluded many of the studies that provided seminal insights into the in vivo functions of PPARs through the use of pharmacological agents; this information is available in other reviews in this volume and elsewhere.

**Figure 1:**
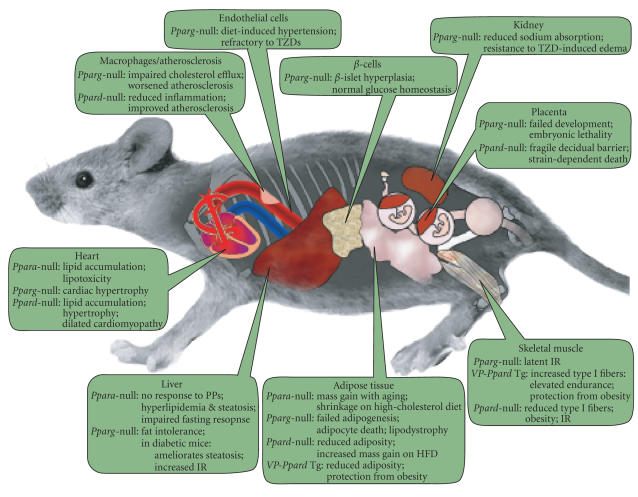
*Genetic manipulations of PPARs—compilation of metabolic phenotypes*. The scheme synthesizes observations from both whole-body and tissue-specific KOs.

It is evident from the studies reviewed that deficiencies or unscheduled expression of PPAR*α*, PPAR*γ*, and PPAR*δ* impact multiple tissues and vital metabolic processes, and that despite their substantial homology and evidence of shared transcriptional targets, the physiological functions of each are unique. These observations are compiled in Figure 1.

Some of the conclusions that emerge from these studies are consistent and irrefutable, such as the critical role of PPAR*α* in the fasting response, the indispensability of PPAR*γ* for adipocyte differentiation and survival, or the role of muscle PPAR*δ* in fiber type determination and basal oxidative metabolism. Other conclusions are solid, but could be refined and extended by further studies; examples include the antiatherosclerotic functions of PPAR*γ*. However, many studies report data and conclusions that seem either overstated or in conflict altogether with other studies. Nevertheless, in case of studies in the latter category we tried our best to summarize the data as published, point out major discrepancies, and where possible, provide plausible explanations for disparities between reports, while leaving it to the readers to formulate their own judgment. Still, the text is likely permeated with some of our own biases, formed through informal discussions with other researchers, familiarity with the evolution of some of the concepts and hypotheses, and our own unpublished work.

As pointed out throughout this review, inconsistencies or erroneous data could readily arise from minor imperfections in the targeting strategy, inappropriate heterogeneity of the genetic background, differences in husbandry, feeding regimens and experimental protocols, and, last but not least, human error. Although these issues need to be ironed out in the long term, one may take the philosophical stance that hard-to-reproduce results are too minor to be biologically significant. This leaves us with the larger, yet-to-be-answered questions that should be addressed by genetic manipulations of PPARs in the near future.

Currently one of the biggest questions concerning PPAR*α* is the therapeutic promise of fibrate drugs and derivatives, which have been all but neglected in recent years. Considering the unique functions of PPAR*α* in lipid clearance and the fasting response, are there adverse metabolic conditions for which the potential of its agonists to provide an ideal treatment has been overlooked? The combined effects of *Ppara* KO and agonists on animal models of various diseases that entail altered lipid homeostasis should provide answers to this question.

For PPAR*γ*, several mysteries beg resolution, none more important than its connection to insulin sensitization, which has thus far eluded definitive proof. The following are three examples for the many potential approaches that could be employed to address this topical issue. First, beyond its importance for adipogenesis and adipocyte viability, is adipocyte PPAR*γ* a major player in systemic metabolism? Can we gain a molecular understanding of the death mechanism of *Pparg*-null adipocytes and use it to delete *Pparg* in these cells while averting their death? Assuming that we can devise such methods to obtain mice with viable *Pparg*-null adipocytes, what would their metabolic phenotype be? Second, we should continue to explore the contributions of PPAR*γ* to metabolic homeostasis through its actions in additional tissues. Considering the critical role of central regulation in energy homeostasis, one glaringly neglected hypothesis is that PPAR*γ* may also function centrally; this idea could be tested by tissue-specific *Pparg* KOs in the CNS and hypothalamic neurons. Third, we do not yet understand the mechanisms of insulin sensitization by *Pparg* haploinsufficiency. Additional in vivo experiments are required to identify the culprit tissue(s) and the target genes whose deregulation underlies this phenomenon.

PPAR*δ* research has been lagging behind that of PPAR*α* and PPAR*γ*, and new findings are starting to trickle from multiple tissue-specific *Ppard* KOs. The immediate significant questions revolve around the detailed mechanistic understanding of PPAR*δ* action in lipid and oxidative metabolism and in inflammation. Considering that *Ppard*-null mice surviving gestation are by and large healthy under standard husbandry, how important are these functions for basal health? And when these functions come into play under metabolic stress, how can they be modulated for the best possible treatment of metabolic diseases? As pointed out above, combination studies of pharmacological agonists and genetically manipulated animals will bring us several steps closer to answering these questions.
